# Supplementary material for: Factors affecting the implementation of evidence-based Progressive Tinnitus Management in Department of Veterans Affairs Medical Centers
Source: PLoS One. 2020 Dec 28;15(12):e0242007. doi: 10.1371/journal.pone.0242007 (PMC7769276; doi:10.1371/journal.pone.0242007)
Supplement: S2 Appendix — (PDF) [file pone.0242007.s002.pdf]

By completing this survey I understand and agree to the following terms:

The PURPOSE of this survey is to obtain information to describe current tinnitus-management practices in VA Audiology and Mental Health programs across the country. This survey should be completed by someone in your clinic who is aware of the clinic's provision of tinnitus services. Completing the survey is voluntary and participants will be anonymous. The survey does not obtain health or sensitive information.

The LONG-TERM GOAL of this project is to implement a comprehensive tinnitus management program that is clinic based and utilizes telehealth modalities to provide these highly needed services to Veterans. To strengthen the national relevance and likely success of our implementation efforts, we have partnered and are working closely with the VA Offices of Audiology and Speech Pathology and Mental Health Services, as well as the VA PT/BRI QUERI (Polytrauma and Blast Related Injuries Quality Enhancement Research Initiative), in the development and conduct of this research.

Informed consent: By completing this survey you are consenting to the terms of this online task. The survey is completely anonymous, i.e., no "personal identifying information" will be obtained. You will be informed at the end of the survey of the potential opportunity to participate in a telephone interview in the following phase of this study, which would require you to send an email message to the Principal Investigator. If you express interest and are selected for a next-phase interview, we will retain your contact information only until the interview is complete. If you express interest and are not selected for the interview (which you would be informed of), we will delete your contact information.

Risks: Study risks are limited to emotional reactions that could result from completing the survey. Although this risk is extremely unlikely, as a participant you do not have to answer any question that causes you discomfort. You can terminate participation at any time for any reason, without consequence. A psychologist will be made available for counseling should the need arise.

Benefits: Although no benefit to the participants is expected, this research could result in improvements to tinnitus services provided to Veteran patients. In this case, many Veterans could benefit from the study.

- ☐ I understand and agree to these terms.

1. **What is your role at your facility?**
   - ☐ Clinical Psychologist
   - ☐ Clinical Social Worker
   - ☐ Other Mental Health Provider

2. **Do you encounter patients who report tinnitus?**
   - ☐ Yes
   - ☐ No

3. **Do you personally provide clinical services specifically for tinnitus?**
   - ☐ Yes
   - ☐ No

4. **Do you feel that your clinic <u>should</u> provide tinnitus-management services to Veteran patients?**
   - ☐ Definitely yes
   - ☐ Probably yes
   - ☐ Probably no
   - ☐ Definitely no

   *Please tell us why you chose this answer:*_____________________

5. **How confident are you that your clinic <u>can</u> provide tinnitus-management services to Veteran patients?**
   - ☐ Very confident
   - ☐ Somewhat confident
   - ☐ Barely confident
   - ☐ Not at all confident

   *Please tell us why you chose this answer:*_____________________

6. **In which VISN do you work?**

Drop down with options from VISNs 1-23

   a. **What is your station number? (first three digits only – no letters)**

7. **In what type of VA facility is your clinic located?**
   - ☐ Hospital
   - ☐ Other ________________________

8. **How convenient is parking for patients?**
   - ☐ Very convenient
   - ☐ Somewhat convenient
   - ☐ Neither convenient nor inconvenient
   - ☐ Somewhat inconvenient
   - ☐ Very inconvenient

9. **How many (<u>total</u>) Mental Health Providers are employed at the clinic where you work?**
   - ☐ 1-5
   - ☐ 6-10
   - ☐ 11-15
   - ☐ 16-20

- ☐ more than 20

10. How many full-time-equivalent (FTE) Mental Health Providers are employed at the clinic where you work?

- ☐ 1-5
- ☐ 6-10
- ☐ 11-15
- ☐ 16-20
- ☐ more than 20

11. What is the approximate average patient volume per day, per full-time-equivalent clinician?

- ☐ Fewer than 5
- ☐ 5-7
- ☐ 8-10
- ☐ 11-13
- ☐ More than 13

12. What days does the clinic where you work normally operate? *Check all that apply*

- ☐ Monday
- ☐ Tuesday
- ☐ Wednesday
- ☐ Thursday
- ☐ Friday
- ☐ Saturday
- ☐ Sunday

13. On the days that your clinic normally operates, what is the average number of hours the clinic is open?

- ☐ **Fewer than 3**
- ☐ **3-5**
- ☐ **6-8**
- ☐ **9-11**
- ☐ **More than 11**

14. Please tell us about your education and/or training specific to tinnitus management.

Graduate program

- ☐ No training specific to tinnitus management
- ☐ 1-4 hours
- ☐ 5-8 hours
- ☐ 9-12 hours
- ☐ 13-16 hours
- ☐ More than 16 hours

Training workshop(s)

- ☐ No training specific to tinnitus management
- ☐ 1-4 hours
- ☐ 5-8 hours
- ☐ 9-12 hours

- ☐ 13-16 hours
- ☐ More than 16 hours

Independent study

- ☐ No independent study specific to tinnitus management
- ☐ 1-4 hours
- ☐ 5-8 hours
- ☐ 9-12 hours
- ☐ 13-16 hours
- ☐ More than 16 hours

Online course(s)

- ☐ No training specific to tinnitus management
- ☐ 1-4 hours
- ☐ 5-8 hours
- ☐ 9-12 hours
- ☐ 13-16 hours
- ☐ More than 16 hours

15. If training in tinnitus management was available and supported by your hospital/supervisor, would you be interested in receiving it?

- ☐ Definitely yes
- ☐ Probably yes
- ☐ Not sure
- ☐ Probably no
- ☐ Definitely no

16. If you had the opportunity to receive training for tinnitus management, which method(s) would interest you? *Check all that apply*

- ☐ Online training
- ☐ Live training at my site
- ☐ Live training at another site (requiring travel)
- ☐ Self-study (guided instruction involving books, articles, etc.)
- ☐ Other: ________________________

17. Clinical supervision is important to follow up training; how would you prefer to receive clinical supervision? *Check all that apply*

- ☐ Direct in-clinic supervision
- ☐ Audio-record my interactions with patients for remote review and feedback by an expert
- ☐ Video-record my interactions with patients for remote review and feedback by an expert
- ☐ Other: ________________________

18. How much time (over a year) would you be willing to invest to receive training in tinnitus management?

- ☐ Up to 1 hour
- ☐ 1-3 hours
- ☐ 3-5 hours
- ☐ 5-10 hours

- ☐ 10-20 hours
- ☐ none

19. Please estimate the percentage of patients in the clinic where you work who report <u>chronic tinnitus</u> (i.e. persistent tinnitus for at least 3-6 months).

- ☐ 0-10%
- ☐ 11-20%
- ☐ 21-30%
- ☐ 31-40%
- ☐ 41-50%
- ☐ 51-60%
- ☐ 61-70%
- ☐ 71-80%
- ☐ 81-90%
- ☐ 91-100%

*[NOTE: any percentage response re: % patients who report chronic tinnitus will generate the same **subquestions**]*

a. <u>Of these</u> [*patients who report chronic tinnitus*], please estimate what percentage report that their tinnitus is **not a problem**.

- ☐ 0-10%
- ☐ 11-20%
- ☐ 21-30%
- ☐ 31-40%
- ☐ 41-50%
- ☐ 51-60%
- ☐ 61-70%
- ☐ 71-80%
- ☐ 81-90%
- ☐ 91-100%

b. <u>Of these</u> [*patients who report chronic tinnitus*], please estimate what percentage report that their tinnitus is a **mild/moderate problem?**

- ☐ 0-10%
- ☐ 11-20%
- ☐ 21-30%
- ☐ 31-40%
- ☐ 41-50%
- ☐ 51-60%
- ☐ 61-70%
- ☐ 71-80%
- ☐ 81-90%
- ☐ 91-100%

c. <u>Of these</u> [*patients who report chronic tinnitus*], please estimate what percentage report that their **tinnitus is a severe/debilitating problem**?

- ☐ 0-10%
- ☐ 11-20%
- ☐ 21-30%
- ☐ 31-40%

- ☐ 41-50%
- ☐ 51-60%
- ☐ 61-70%
- ☐ 71-80%
- ☐ 81-90%
- ☐ 91-100%

d. Of these [*patients who report chronic tinnitus*], please estimate what percentage **request services to help their tinnitus?**

- ☐ 0-10%
- ☐ 11-20%
- ☐ 21-30%
- ☐ 31-40%
- ☐ 41-50%
- ☐ 51-60%
- ☐ 61-70%
- ☐ 71-80%
- ☐ 81-90%
- ☐ 91-100%

e. Of these [*patients who report chronic tinnitus*], please estimate what percentage report a **sound tolerance** problem (when sound is too loud or annoying while tolerated comfortably by other people)?

- ☐ 0-10%
- ☐ 11-20%
- ☐ 21-30%
- ☐ 31-40%
- ☐ 41-50%
- ☐ 51-60%
- ☐ 61-70%
- ☐ 71-80%
- ☐ 81-90%
- ☐ 91-100%

20. Please estimate how many patients are referred monthly from the clinic where you work to another clinic (such as ENT, Primary Care, Audiology Clinic) specifically for tinnitus services?

- ☐ None
- ☐ 1-10
- ☐ 11-20
- ☐ 21-30
- ☐ 31-40
- ☐ 41-50
- ☐ More than 50

21. Please estimate how many patients are referred monthly to the clinic where you work from another clinic (such as ENT, Primary Care, Audiology Clinic) specifically for tinnitus services?

- ☐ None
- ☐ 1-10
- ☐ 11-20

- ☐ 21-30
- ☐ 31-40
- ☐ 41-50
- ☐ More than 50

22. Are any tinnitus-specific questionnaires used in the clinic where you work?

- ☐ No (<u>go to #23</u>)
- ☐ Yes

  (if checked yes)
  - ☐ Tinnitus Handicap Inventory (THI)
  - ☐ Tinnitus Functional Index (TFI)
  - ☐ Tinnitus Reaction Questionnaire (TRQ)
  - ☐ Tinnitus and Hearing Survey (THS)
  - ☐ Self-Efficacy for Managing Reactions to Tinnitus (SMRT)
  - ☐ Other __________________

23. When patients request services specific to tinnitus, which special services and/or devices do clinicians at your site provide? *Check all that apply*

- ☐ Cognitive Behavioral Therapy for tinnitus
- ☐ Acceptance and Commitment Therapy for tinnitus
- ☐ Mindfulness based counseling for tinnitus
- ☐ Biofeedback for tinnitus
- ☐ Progressive Tinnitus Management (PTM)
- ☐ Other ______________________________

24. What would you say are some of the main challenges to using tinnitus management services at your facility?" *Please type your response here*

____

____

25. Is specialized telehealth equipment currently available at your site?

- ☐ No (<u>go to #26</u>)
- ☐ Yes

If yes to #25:

a. Which equipment is available? *Check all that apply*

- ☐ Clinical Video Telehealth equipment
- ☐ Home Telehealth equipment

b. Does the clinic where you work offer tinnitus services via telehealth?

- ☐ No
- ☐ Yes

c. Does the clinic where you work offer mental health services that are not related to tinnitus via telehealth?

- ☐ No
- ☐ Yes

26. Is Progressive Tinnitus Management (PTM), or services based on PTM, available at the clinic where you work?
- ☐ No (go to #27)
- ☐ Yes

If yes to #26:

a. **Are patients referred to the clinic where you work according to the PTM Level 1 Triage guidelines?**
- ☐ No
- ☐ Yes

b. **Do patients complete questionnaires as part of the PTM Level 2 Audiologic Evaluation?**
- ☐ No
- ☐ Yes

c. **Do patients with bothersome tinnitus attend PTM Level 3 Group Education?**
- ☐ No
- ☐ Yes

d. **Is PTM Level 4 Interdisciplinary Evaluation an option for patients if needed?**
- ☐ No (go to e)
- ☐ Yes

If yes to d:

i. **Is the evaluation performed by:**
- ☐ An Audiologist
- ☐ A Mental Health Provider
- ☐ Both
- ☐ Other____________

e. **Is PTM Level 5 Individualized Support an option for patients if needed?**
- ☐ No (go to #27)
- ☐ Yes

If yes to e:

i. **What services are offered for Level 5?** *Check all that apply*
- ☐ One-on-one intervention with Mental Health Provider
- ☐ One-on-one intervention with Audiologist
- ☐ Other________________________________

27. We need your help so that we can do a better job of helping our Veterans with tinnitus. Are you willing to be interviewed to answer questions about (1) your perceptions of PTM (e.g., whether it's evidence based; if you think it can help your patients); and (2) perceived barriers and facilitators to PTM (e.g., patient needs/resources; policies/incentives; implementation climate such as clinical space, time, and staff training)? (telephone interview will require approximately 30 minutes) If so, please email Dr. James Henry at james.henry@va.gov to be scheduled for an interview about challenges and facilitators to PTM.

Thank you for your participation!
