# Supplementary material for: Factors affecting the implementation of evidence-based Progressive Tinnitus Management in Department of Veterans Affairs Medical Centers
Source: PLoS One. 2020 Dec 28;15(12):e0242007. doi: 10.1371/journal.pone.0242007 (PMC7769276; doi:10.1371/journal.pone.0242007)
Supplement: S2 Dataset — Responses to open-ended survey questions used in qualitative analysis. Blank (i.e. missing) responses have been excluded. Responses for each survey item are presented in a randomized order, organized by discipline. Text that has been changed in order to de-identify the respondent is indicated in [square brackets], any text that has been removed for de-identification purposes is indicated by square brackets with an ellipsis […]. Typos in responses have been indicated by (sic). (DOCX) [file pone.0242007.s004.docx]

**Caption**: Blank (i.e. missing) responses have been excluded. Responses for each survey item are presented in a randomized order, organized by discipline. Text that has been changed in order to de-identify the respondent is indicated in [square brackets], any text that has been removed for de-identification purposes is indicated by square brackets with an ellipsis […]. Typos in responses have been indicated by (sic).

***Following the question “Do you think your clinic should provide tinnitus-management services to Veteran patients?” (Q4) survey respondents were asked “Please tell us why you chose this answer.”***

| ***Audiology Survey responses*** |
| --- |
| It is a condition that causes many problems especially with our PTSD/TBI vets. Helping them with tinnitus management improves their quality of life. |
| Tinnitus is a common complaint among our clinic population |
| It is a needed service. |
| Tinnitus is a common complaint, for many it is extremely bothersome. Any relief we can give the patient is greatly appreciate (sic) by the veteran. |
| Because the prevalence of tinnitus sufferers are increasing significantly and I would like to be able to provide/or attempt to provide some relief. |
| Tinnitus is a common problem that affects many veterans' quality of life, and we now have an effective and useful treatment program in PTM. |
| This is in our area of specialty and we have the means to offer assistance via our PTM program and TSG devices. |
| Veteran's deserve and derive benefit from these services. The PTM program is significantly better than what was available when I first began my career, which was basically education about causes and ways to introducenoise (sic) into the environment. |
| Tinnitus is often associated with hearing loss. |
| it is part of hearing health management, and a primary complaint of many Veterans |
| Tinnitus management is a very sought after service at the VA. |
| my short answer would be ' who is more qualified to provide service with us'. Of course I also think tinnitus can be a very disabling condition in certain people |
| This is a needed service, however, I fee (sic) that data needs to be collected on the incoming 'baby boomer' population to tease out truly dibilitating tinitus (sic) from those seeking service connection determination or elevation. |
| Though the majority of patients who present with tinnitus in our clinic are happy with some education and information on the use of soothing sounds in their environments, there are those that occasionally present with more difficulty coping with their tinnitus. I do think Mental Health should be involved but we have not achieved that very well here. |
| Progressive Tinnitus Management should be incorporated for VEterans (sic) who expereience (sic) bothersome tinnitus, but our facility does not currenlty (sic) have the resources/procedures in place to provide these services to an in depth extent. |
| Many veteran report tinnitus, and many report they suffer from it. |
| Shortage of staff to implement the program. |
| Tinnitus is very common and bothersome to many of my patients, so having a treatment protocol for tinnitus is vital. |
| Most prevalent SC disability. |
| Many patients primary complaint is tinnitus. |
| It is within our scope of practice. It is inappropriate to pretend tinnitus is not a problem. |
| To address patient concerns and educate the patient on tinnitus |
| I feel that we should provide tinnitus management services because sometimes the Veteran needs more than a 5 minute counseling session at the end of the hearing test appointment. Tinnitus management allows for comprehensive teaching on tinnitus and how to cope with it. I think it is great. |
| Major issue for a large percentage of our patients. |
| TO PROVIDE THE BROADEST RANGE OF SERVICES POSSIBLE. |
| Tinnitus has been the #1 or #2 SC disability of all Veterans for the past several years. |
| This symptoms (sic) is reported by a large amount of Veterans that for some is bothersome enough to disturb their activities. |
| Tinnitus is a highly prevalent condition in this population and many veterans seek information regarding management and resources. |
| Education about tinnitus is the first step for management of tinnitus. Addressing the emotional aspect and educating Veterans about tinnitus help validate their concerns and many feel better about their tinnitus. Others need more discussion which is where PTM can provide further help. |
| We already provide PTM in the clinic |
| use of maskers bedside device hearing aids |
| The need for this service is increasing. |
| Tinnitus is a growing concern among veterans. |
| As one of the top service connected disabilities in all of VA healthcare, it should be part of an Audiology Clinic's Mission Statement to provide tinnitus management, coordinate interdisciplinary care and advocate for the Veteran's affliction. |
| Tinnitus is a common complaint among veterans. We are obligated to help them. |
| With the number of TBI cases resulting in younger veterans with tinnitus in the absence of hearing loss, it is imperative that we offer assistance to this group of veterans. |
| Because tinnitus is a real problem! |
| We should provide tinnitus-management services to our veterans because there is a great need for them. Tinnitus is the number one service connected VA disability. Audiologists are the most appropriate providers for tinnitus management. |
| I provide those services for tinnitus management to improve veteran's lives |
| Tinnitus is the second most service connected disability at the VA, it can disrupt people's lives and affect their quality of life. |
| We have seen a marked increase in pt's whose primary complaint is bothersome to debilitating tinnitus. I want to provide the appropriate care for this rising population for whom this sx interferes with their lives. |
| need to help vets manage their lives and teach them how to live wiht (sic) tinnitus |
| Total care |
| We currently provide tinnitus management service. It is definitely a role for audiologists |
| Tinnitus is commonly reported to the audiology staff as a presenting problem. |
| Tinnitus is the #1 most service connected disability, therefore almost all my patients report problems with tinnitus. |
| It is a disorder afflicting Veterans that must be addressed. |
| Tinnitus management services are needed for veterans that are bothered by their tinnitus. |
| It is a need that our veterans have, but we have not been able to expand our services to provide it due to lack of support from senior management. |
| Tinnitus being the #1 disability claim, with all its associated psychological consequences needs professional attention. |
| Audiologists are uniquely qualified to counsel veterans on tinnitus management at the first contact. |
| Tinnitus is a problem for veterans just as hearing loss is an issue for them. |
| It is a common problem and veterans ask for assistance in management of their Tinnitus. |
| Tinnitus is a growing problem among our patients. It affects every aspect of their lives. It is our responsibility as their caregivers to address the problems that affect them on a daily basis. |
| Provision of tinnitus causes and management options are generally very helpful for the veterans. |
| tinnitus is one of the top disabilities in the VA |
| Tinnitus is a complaint of most of our Veterans. I believe we should be providing at least a minimum of counseling and issuing of devices in helping them manage their tinnitus. |
| A large percentage of our patients experience tinnitus. For some of these patients tinnitus is very bothersome and education and management for tinnitus for these patients is desired. |
| This is a common reason for veteran referral. |
| A large percentage of our patients experience tinnitus, some of them to a very bothersome degree. Education and tinnitus management is desired by many of the patients. |
| There are Veterans who we seen who find their tinnitus bothersome and need support to find relief. |
| TINNITUS MANAGEMENT IS PART OF OUR SCOPE OF PRACTICE AS MUCH AS HEARING LOSS. |
| So many are bothered by tinnitus and need some guidance on coping strategies. |
| Overwhelming number of veterans present with tinnitus. |
| Tinnitus is a top service connected condition and we should do what we can to help treat it. |
| So that they can be better educated about their condition and learn ways to help them better deal with it. |
| It's an extremly (sic) common problem in our pt. population. |
| This is a very large concern for our patients at this clinic. |
| It is an obvious need in the clinic. |
| Many patients report tinnitus |
| We see a lot of vets who suffer from TBI (we have an in-patient TBI program) who also have significant difficulties with tinnitus. |
| High % in this population w/it. |
| We provide tinnitus management education and work closely with mental health providers, ENT, pharmacy as well other providers when needed. |
| unaddressed on every level here |
| It isoften (sic) a primary complaint of veterans. They have so often been told there's nothing that can be done, but I don't think this is true. |
| Many combat veterans report history of tinnitus, which they say is interferring (sic) with communication and sleep. Many report that it causes undo stress. |
| Because it's one of the top two SC disabilites (sic) in the VA and early intervention has been shown to help in the long run. |
| Tinnitus patients need assistance in dealing with a very bothersome symptom. They ask often for help and we need a strong program that will offer them the relief they seek. |
| If they have a hearing loss we fit with hearing aids, we chose those that have maskers to be used if needed. If they don't have a hearing loss we counsel |
| We have many veteran (sic) that have bothersome tinnitus. Additionally since tinnitus is one of the top service connected disabilities how would we not provided management services to veterans. |
| We have patients that can benefit from tinnitus management. |

PTSD = Post-Traumatic Stress Disorder

TBI = Traumatic Brain Injury

PTM = Progressive Tinnitus Management

TSG = Tinnitus Sound Generator

SC = Service Connected

VA = Veterans Affairs

ENT = Ear, Nose and Throat

| ***Mental Health Survey responses*** |
| --- |
| Tinnitus is disruptive to the lives of some veterans; addressing it may help reduce this disruption. |
| Appropriate communication, so that Veteran fully understands discussion, instructions, etc. |
| Some veterans report it is an annoying experience during their daily lives. |
| to evaluate the contribution of tinnitus to other psychiatric issues, eg depression as well as to be able to consider tinnitus in the differential diagnosis of other auditory perceptual disturbances, eg auditory halluicnations (sic) |
| because psychiatrists are not trained to provide this type of service. we have an excellent audiology dept here that does a wonderful job providing these services |
| I know that there are current research efforts regarding tinnitus, but am not aware of what the updates are, especially regarding mental health interventions. Especially if this involves specific neurocognitive techniques. |
| That is really not within the scope of mental health. |
| I am unsure of the number of patients in my service who have tinnitus |
| because primary care deals with tinnitus, it is not a frequent enough occurrence in our population to warrant tinnitus-management services |
| Area which does not get addressed adequately |
| Tinnitus can be very stressful and it affects the patient's sleep hygiene- depending upon the severity, it can be disabling. |
| Because of how prevalent the problem is. |
| should be better managed by Audiology |
| Most of our Veterans suffer from tinnitus and has 0 treatment for the problem. |
| As a responce (sic) to care for all service related needs, tinnitus should be part of those needs. |
| no available service exists at our clinic |
| am not trained to manage tinnitus...if bothersome for veteran, I refer out to audiology for eval/management |
| My staff and I see veterans who have service connection for tinnitis (sic) but veterans have nor t specicifically (sic) asked us to participate in their care regarding this |
| Tinnitus is a frequent additional complaint for Veterans I see. However, most often tinnitus is not the primary focus of interventions when other more pressing mental health or physical injuries predominate. |
| Due to the number who experience and the negative imapct (sic) on their mood, difficulty adjusting to the loss of physical functioning |
| It impacts all aspects of life |
| NOT SURE WHAT MENTAL HEALTH CAN OFFER |
| I beleive (sic) the problem is more common that we realize. I'm sure Vets who come to MH appointments don't being up the issue even though it is distressing; they may feel there is nothing MH can do. |
| So many veterans suffer from Tinnitus and do not know what to do about it |
| I have used benzodiazepines and addressed in therapy in the past but have referred to ENT via primary care when needed |
| Tinnitus is a chronic condition that interferes with quality of life and can influence moods and anxiety |
| Tinnitus can be very debilitating |
| High percentage of vets have tinnitus |
| tinnitus requires specialty care |
| Maybe a needed service |
| Tinnitus is very common in out patient population and it causes some of the patients distress |
| I think we should provide help with whatever ailment a veteran has, or needs help with. |
| such a large percentage of vets claim to have this difficulty. It clearly affects the potential to influence them, not only because of their distractedness but also due to the annoying, painful, discouraging aspects of the difficulties |
| Common condition (sic) |
| Veteran's suffer greatly with tinnitus and mental health specialists are in a position to offer skills training, mental health services that are often needed and a forum to discuss challenges with other Veterans. |
| we provide social work case management and refer to audiology for this specialty service |
| It would be nice to have these services, but our clinic is so tiny that I doubt we could provide them unless we move to a larger facility. |
| common problem |
| tinnitus is not generally defined as a mental disorder |
| Tinnitus is not a DSM 5 disorder. |
| Some are significantly distressed by this symptom. Some tools or specific referrals might prove very helpful. |
| We are 4 hours from the nearest VA medical center. |
| its a problem they report |
| It may be outside a psychologist's scope of practice |
| Many Veterans report Tinnitus related psychological distress and life interference, thus providing services that can address these issues would be extremely beneficial for overall health and well-being. In our clinic's preliminary stages of implementing a [tinnitus] program, we have observed significant improvements in psychological distress and Tinnitus related interference. |
| We should take care of the medical problems of our patients. If a pt presents with tinnitus we ought to be able to treat that person. |
| We focus on mental health services and can refer for specialty car (sic) |
| Small # |
| Provider does not have the clinical skill to assess and manage tinnitus |
| We could work with Audiology to help with education, management, and habituation, etc... as well as reviewing possible causes (sleep deprivation, etc...) |
| Many veterans have tinnitus and need the services that mental health providers offer. Many veterans with tinnitus also have comorbid mental health symptoms such as depression and/or anxiety. |
| I would like to see it provided in audiology, but I would be willing to see it provided here if it is not going to be conducted elsewhere. |
| There appears to be a relationship between the level of anxiety and the intensity of the tinnitis (sic) in several of my patients. Although the tinnitus (sic) never goes away, it appears that once the anxiety is better managed the tinnitus (sic) is, as well. |
| I'm not sure if we would have the time to add a new program into our system. We are already behind in trying to meet national standards for other services. |
| I will discuss with veterans their tinnitus but never trained so training on what works could be beneficial |
| I know there there (sic) are excellent therapies such as CBT that have been effective in reducing tinnitus symptoms. |
| Tinnitus can exasperate mental health symptoms and vice versa. |
| This is a common issue for our Service Members and we should serve them as a whole not just parts and peices (sic). |
| My background is in the field of Deafness and has expanded to include late-deafness and veterans with tinnitus. These areas are the highest service-connected areas for our veterans and I work with this populatoin (sic) on a regular basis. Sadly, not many professionals are trained in this area but I am trying to educate others, including interns and residents. |
| I am aware that there are some behavioral/psychological protocols for ameliorating the symptoms of tinnitus, my clinic is the Mental Health Clinic. |
| Its a common symptom associated with TBI. |
| I experience tinnitus nearly every day and am service connected for the issue. I have not recieved (sic) any assistance with this since it was diagnosed while active duty. My provider has never asked about it beyond my first appointment in [year]. |
| A number of veterans suffer from this condition and it does impact mental health. However, deafness and hearing loss is one of my specializations so I do recognize the need for services. I'm constantly on a soapbox about it but I can't say how much attention is paid to this area. |

MH = Mental Health

ENT = Ear, Nose and Throat

DSM 5 = Diagnostic and Statistical Manual of Mental Health Disorders (Fifth Edition)

VA = Veterans Affairs

CBT = Cognitive Behavioral Therapy

TBI = Traumatic Brain Injury

***Following the question “How confident are you that your clinic can provide tinnitus-management services to Veteran patients?” (Q5) survey respondents were asked “Please tell us why you chose this answer.”***

| ***Audiology Survey responses*** |
| --- |
| Had training in various treatment options |
| We have the PTM tools available to us. |
| We are constantly changing and improving our tinnitus management clinic, we review and ridesign (sic) our class, hold meeting with our conctat (sic) mental health provider and offer veterans help when needed. They feel free to contact us and letters are also sent to remind them that if needed more help providers are available. |
| I know that some providers are capable but I do not feel all providers are as strong in the services they provide. The level of care an individual with tinnitus receives is really 'luck of the draw' in the clnic (sic). Some providers are aware of research and are evidence based in their practice and others aviod (sic) addressing tinnitus at all. |
| We have been operating a PTM program for about one year with good success. |
| Because there are many tools available to VA audiologist to assist in the goal. |
| We have trained staff on PTM, participate in tinnitus research, and all clinical audiologists at out facility have an interest in tinnitus management to best help our patients. |
| We have attended PTM trainings in person, used TMS modules, watched DVDs, and we have been conducting PTM since [date] |
| We have a tinnitus management team in place to care for these pts. This program has been developed in conjunction with our MHSL and is co-led by a psychologist who specializes in chronic pain management. |
| Not all Audiologists at this facility see the need to provide anything more than a brochure on what it is. Indeed this is enough for some but not all. |
| It could be time consuming and also tinnitus management handbook is not available sometimes. |
| I have taken over 30 hours of continuing education in tinnitus management |
| We provide a tinnitus management class |
| We have a tinnitus management program but hard to get Vets to participate. |
| I run the PTM sessions at the hospital |
| Training |
| We do individual counseling but do not provide group PTM right now. We do refer to psychology for CBT as necessary. |
| We have tinnitus classes each month taught by audiology and psychology. We offer one-on-one counseling and treatment for tinnitus. |
| we offer ptm (sic) classes and sound devices to veterans |
| We have a Tinnitus Clinic in which we follow guidelines set for by PTM. |
| We all seem to be starting the approach of how to work with tinnitus patients differently. |
| We have used Neuromonics in the past with little success. We no longer use Neuromonics. We issue the Marsona kits, customized to each patient's set of complaints and ear level maskers. But for those who really struggle with tinnitus, especially those with PTSD, I don't know that we adequately address all the issues with any success. |
| We have a well trained tinnitus provider who does group clinics and has come from another VA that did group clinics in conjunction with psychology. Only here we are struggling to get anyone in Psychology involved and we feel is would benefit our patients. |
| We currently provide services |
| My staff is generally competent, however, we are still 6 to 12 months from obtaining steady state on basic services after many years of understaffing. |
| We are already providing PTM services |
| we offer a range of device from bedside noise generators, to hearing aid maskers to the Nueromonics device. we do not offer any cognitive therapy however |
| We've been doing it for several years, albeit w/ mixed results. |
| We are implementing PTM in our clinic. |
| Our providers are adequately trained. Our problem is having the time in our schedules to set up clinics specifically for tinnitus management. |
| We are already providing tinnitus management services, and they are explanding (sic) |
| We provide PATM, additional counseling, and use of ear-level noise generators. |
| We have not developed a comprehensive tinnitus management program but manage individual sufferers as best as we can with the resources (e.g. tinnitus workbook, tinnitus sound generators, etc. etc). |
| Experience in dealing with tinnitus patients for many years and the develop of PTM programs and other programs have proven at this clinic to provide Veterans with the assistance they seek. |
| Well trained staff |
| We offer telehealth program for tinnitus patients. |
| BECUASE i DO IT AND i CAN SEE THE EFFECTS OF OUR ASSISTANCE. |
| I am the provider for the PTM program |
| We have a PTM program that has been successfully up and running since January 2013. We have served a large number of tinnitus patients and continue to do so. |
| Space is an issue here and not the best set up for groups. |
| Many of our audiologists have many years of experience working with tinnitus. Most of the patients in this clinic report tinnitus with their hearing loss. It is a perfect place to provide these services. |
| May add staff. |
| I AM NOT SURE OF WHAT SUPPORT WE CAN EXPECT FROM OTHER AREAS SUCH AS BHC. |
| 'All of us have a diferent (sic) approach and is not necesarily (sic) based in clinical or research fundaments. Some of use don (sic) even follow the clinical practices provided'. |
| There are no services currently in place, other than providing a self-help workbook. |
| We have clinicians that are well-equipped and willing. However, our lack of support from senior management is a hindrance. |
| Tinnitus cases are often complex and the treatment options are varied and not clearly defined. If clinicians only see a few cases now and then they are not confident in handling these cases. So we need more clear cut treatment plans and direction regarding these services. Sometimes mental health issues, PTSD, etc., impact tinnitus management and Audiologists may feel some issues encountered are out of our scope of practice and/or comfort zone. |
| Our clinic is knowledgable (sic) in the area of tinnitus, but lacks the manpower to be able to add an additional focus at this time. |
| We have a tinnitus management protocol which is a modified PTM that, according to local data, has been proven to be effective at reducing veteran's awareness and annoyance of their tinnitus using pre- and post-education/sound therapy counseling Tinnitus Reaction Questionnaire scores. |
| we do the best we can with options available for tinnitus management. We could all improve on other strategies or processes in the future |
| we have one audioolgist (sic) who has taken the lead in creating a program for Veterans with tinnitus, including clinical treatment, staff education (our service and others), and program development. |
| We see tinnitus patients but not in great numbers. |
| We have the support of our service chief and are motivated to develop our PTM program. |
| Implementation of a tinnitus program will require more structure to our clinic schedule than we currently have. Adding structure at this point may be a challenge. |
| The VA Healthcare system provides an excellent framework of support to audiologists providing tinnitus management through the Progressive Tinnitus Management System (PTM). The VHA has resources that any audiologist can rely on to provide state of the art tinnitus management. |
| i already provide these services |
| it is not important to people here |
| I am very confident because I have sat in on tinnitus management and seen patients who have gone through the program. I feel that they are getting the care and treatment that they need, and they have told me on several occasions, how helpful they thought the program was. |
| Already providing tinnitus management services such as TRT and Neuromonics Trained through Dr Jastreboff |
| Implementing new processes and procedures is a very difficult process within the system and implementation would likely fall second to improving access for current services. |
| We have a good program in place, people who are caring and interested, involvement from other specialities (sic) |
| I have tinnitus myself and feel patient because I understand how frustrating it can be, but also there usually is a $ associated with tinnitus so I feel that some allow it to be more bothersome (or say it is) for the benefit they would obtain. |
| We have 1 audiologist dedicated to tinnitus management and we are providing a modified version of PTM for our patients. Every audiologist is knowledgeable and we all provide counseling as well as devices to manage tinnitus. We also have a recreational therapist who provides relaxation therapy. |
| I have staff audiologist trained and dedicated to manage this symptoms |
| Provide in person and telehealth classes for tinnitus |
| We are familitar (sic) with tinnitus management services. |
| The staff are up to date on tinnitus management concepts. |
| We are already providing a PTM program. |
| I have been doing PTM for 6 years already. |
| We have an active program in place, PTM, classes with follow up as needed. |
| We don't have the resources or space to provide certain aspects of PTM such as the group sessions. |
| We currently provide PTM in collaboration with a psychologist from the Mental Health department. The majority (>90%) of veterans who complete the group education sessions report the sessions were informative and helpful in learning to cope with their tinnitus. |
| We have a Tinnitus Management clinic in place that follows the guidelines set forth by PTM |
| There is only one audiologist that has had tiinitus (sic) training. |

PTM = Progressive Tinnitus Management

VA = Veterans Affairs

TMS = Talent Management System

MHSL = Mental Health Service Line

CBT = Cognitive Behavioral Therapy

PTSD = Post-Traumatic Stress Disorder

PATM = Progressive Audiologic Tinnitus Management

BHC = Behavioral Health Clinic

VHA = Veterans Health Administration

TRT = Tinnitus Retraining Therapy

| ***Mental Health Survey responses*** |
| --- |
| I am confident our staff could provide good tinnitus management; however, we have staff shortages at our clinic and are already stretched thin. |
| I don't think this is something that mental health providers in general feel well equipped to address. |
| I believe that we take care of most things. I would assume that our physicians can do this work as well. |
| We have good people here, but it is not something that seems to be on the radar. |
| Not trained in any current advances. |
| I do not believe that anyone in my clinic has been trained in any of these protocols. |
| I work in PC-MHI. This is a natural place to provide intervention for medical concerns that can be assisted by behavioral interventions. Our providers are astute and motived. I'm confident they can quickly pick up needed intervention skills with some targeted training. |
| lack of EBT's in this area |
| Again do not know of the impact |
| I am unclear what the treatment is so I cannot answer this fully. I would need further education on this subject. |
| Other than general relaxation and mindfulness strategies, I don't think anyone is specifically trained to do this. However, we could conduct services after some training (i.e., workshop). |
| Mental health staff are not usually familiar with techniques to address tinnitus. |
| I am not aware of any staff with expertise in this area. |
| Not trained to treat medical conditions |
| supportive therapy likely the only treatment/possibly medication |
| We are already overwhelmed, and this would likely require additional training, etc... |
| not enough staff |
| Providers do not have the clinical skill to assess and manage tinnitus |
| we have no specialist in mental health |
| Our current [tinnitus] program has expanded with each cycle and Veterans seem to appreciate that there is an available service offered. |
| Already pressured to provide EBP, complete all behavioral health screens, access performance measures, medication management etc.... |
| Space issues. |
| too small of a CBOC |
| I don't know if they provide services or not. |
| We are currently offering such services in conjuction (sic) with audiology and it works really well to do so. |
| We have providers who could learn how to treat and help Veterans suffering. |
| No training or experience |
| Behavioral interventions are not well understood. |
| need commitment and trianing (sic) of audiology staff; limited psychological staff available |
| We are Mental Health and we currently do not provide any services |
| We have highly trained psychologist here |
| It is difficult to implement new programming at this clinic |
| Extremely under staffed for our ever increasing work load |
| Staff may not recognize this to be as serious a problem as PTSD or other Major mental health condtions (sic). |
| Tinnitus is not always a VBA-rated disability and as such patients encounter specialty service copays ($50/session) for services delivered by Polytrauma/TBI providers and standard copays ($15/session) for general mental health providers. |
| I do not know what services are available and have been shown to be effective. |
| we probally couls (sic) however more concerned that training wouldn't be provided |
| tinnitus requires specialty care |
| Difficult to treat |
| Unfamiliar with this clinic's management of tinnitus. |
| see above |
| Can not make this decision because I don't know what tinnitus-management services are. |
| no scope of practice |
| I am basing my answer of 'Barely Confident' in that providers are spread very thin. Although I am constantly on my 'soupbox' (sic) about this issue, I'm not sure it can take as a high a priority as other services. I would like to see this change!!! In addiition (sic), I did start to provide PTM services a few years ago with an Audiologist. We were both limited in the amount of time we were able to devote to the service and I was then detailed to another service. Now, I try to incorporate using parts of your manual with individual veterans. In addition, I arranged for our Audiology to present a seminar on this topic to interns and residents. |
| we dont have the speciality (sic) |
| hard to treat |
| As stated above, I don't believe many are trained or aware of the importance of this type of service. Although I try to get info out to staff. |
| I don't feel our staff have the training to address these issues. |
| Out of my scope of expertise. |
| It shouldn't be that hard and they may already be doing it and I just don't know. I only work night shift on the weekends. |
| Our leadership supports interdisciplinary care and seems to value this service. |
| Lack of training for staff |
| My clinic is a behavior healht (sic) clinic |
| because we are not |
| we are not trained to do so we do not have audiology services on our campus |
| I am unaware of the treatments and proper ways to care for tinnitus. |
| I am not yet familiar with treatment protocols for this. I did recently hear about a treatment protocol and am looking into this. |

PC-MHI = Primary Care - Mental Health Integration

EBT = Evidence-Based Training

EBP = Evidence-Based Practice

CBOC = Community-Based Outpatient Clinic

PTSD = Post-Traumatic Stress Disorder

VBA = Veterans Benefits Administration

TBI = Traumatic Brain Injury

PTM = Progressive Tinnitus Management

***What would you say are some of the main challenges to using tinnitus management services at your facility? (Q25 in Audiology Survey, Q24 in Mental Health Survey)***

| ***Audiology Survey responses*** |
| --- |
| scheduling time |
| getting the patients to show up for groups |
| Tai-chi |
| scheduling, time |
| The only challenge our program is currently facing is that patients are having to wait 4-5 months to attend PTM workshops because they are very popular. |
| Limited time available to spend with veterans. No formal program available due to limited resources and space. |
| Space, parking, coordination of services, and time. |
| Getting veteran's to comitt (sic) to a 5 week course (1 hr per week); distance traveled for appts (we also offer via tele-med) |
| Many pts live some distance from the facility. Both traffic and parking are significant problems. |
| Lack of space to do group counseling, requiring one on one counseling. Lack of training for some Audiologists. |
| Sometime patients do not come to follow up visits. Sometime patients that do come to follow up visits have not read the handbook or do the sound plan. |
| not enough staff to provide the level of service I would like. |
| Many times veteran do not show up for the class and it is difficult to find space to hold the class. |
| Veterans do not stay in program. Only a few truly want help for it while others only want to be service connected for it. |
| Letting other clinics know that we have tinnitus programs. |
| Time |
| Staff and time |
| currently, tinnitus services are at the medical center not CBOCs |
| lack of mental health professionals involved in ptm (sic). |
| Patient compliance with completing assigned workbook chapters/workbook activities. |
| no formal training |
| Commitment of patients to follow through, to return for appointments and to get other disciplines involved such as mental health. |
| Not enough providers doing it. |
| Time and space |
| we are still backlogged on basic diagnostice (sic) and have very little space to dedicate to counseling |
| PTM is offered but not many choose to attend. |
| we truly have no true counseling program and dont (sic) appear to have any mental health experts who are aware enough of tinnitus (despite our educational outreaches) to feel comfortable talking about it |
| Challenging pt. population, very often w/ significant concomitant psych and other issues. |
| I was able to promote one of my staff to be a GS 13 Specialized Clinic Program Manager for our Tinnitus Program. She conducts weekly PTM classes and is an asset to our service. Not all patients can make the […] classes though so they are scheduled individually other days with myself or her accordingly. Challenge is no more PTM workbooks from EES that are used for our PTM classes. |
| Time |
| scheduling (coordinating clinics), the adminitstrative (sic) work involved that falls mostly on the audiologist heading the program. |
| having all clinicians trained |
| Difficulty with follow-up due to the demand of overall audiology services and small staff numbers. |
| Time |
| N/A |
| patients think we can 'cure' their tinnitus and don't always follow through on their own |
| Time constraints |
| The two group session deters patients from signing up. More likely to come to one group session. |
| None |
| Time constraints. |
| GETTING RESOURCES FROM OUTSIDE AUDIOLOGY SUCH AS FROM BEHAVIORAL HEALTH FOR COGNITIVE RETRAINING. |
| Clinicians does not have the expertise to provide information regarding emotional aspect of tinnitus (sic). They are refered (sic) on this aspect to the clinic from mental health because we are the ones that treated. Patient have other issues that are not replated (sic) to tinnitus that need to be resolved not by an audiologist. Audiologist and Mental health need education and a sharing agreement, work together to provide the services. Health care providers such as PC physicians and mental health and ENT need more education. |
| lack of training. |
| Senior management allowing us to expand our services. They do not support it based on clinic access. |
| Clear cut treatment plans and protocols. Seeing enough of these patients to gain confidence in treatment skills. |
| Lack of time & recent training |
| high no-show rate for tinnitus follow-up appointments, lack of mental health support (for cognitive behavioral therapy or for those patients with unrealistic expectations) |
| no measure to determine objectively if what we recommend is working and what percentage of patients using device are satisfied |
| Demands of busy clinical practice leaving less time to devot (sic) to subspecialty such as tinnitus management. |
| Staffing |
| prosthetics department will not approve requests for SoundPillows, Neuromonics/SoundCure (for those that received little benefit from hearing aids/ear level sound generators and counseling). It is also difficult to get all of the audiologists on the same page with regards to treating tinnitus. |
| 1. Our 2 audiologists who specialized in tinnitus management have transferred to other clinics. 2. Pressure from upper management to see more patients everyday. |
| The number one issue is staffing and clinical time. Level 4 & 5 patients (PTM) require multiple appointments and at time coordinated care with mental health. I feel we have the best resources available as a profession, but as usual, I would love to have a Grade 13 supervisory tinnitus audiologist. |
| time to devote |
| staff |
| Patients are unwilling to attend group sessions. No direct liaison with behavioral health. |
| I do not think we have a lot of challenges, I believe, in general, we are able to meet the Veteran's needs for tinnitus management (sic). |
| Patient commitment to the programs |
| Time constraints, lack of training available for providers, lack of communication between departments. |
| number of patients difficulty with continuity of care among providers both within Audiology Service and across to other specialties high staff turn over in depts other than Audiology make it difficulty to educate them on their role |
| time, distance the pt has to travel cuts out regular formal management, materials to give to the pts (ran out of PTM books), |
| Having a multidisciplinary approach. Getting patients to agree to return for our [tinnitus management] class. We hope to implement this as part of our new Tele-health clinics. |
| Length of program. Time factors. Not enough clinical staff. |
| Proper follow up with telehealth. Difficulty having mental health provide full services |
| We use some concepts of PTM. |
| We use tinnitus management services at this facility. We refer to behavioral health when the above tools do not suffice. |
| By only offering the workshop once a week it sometimes precludes veterans with travel issues from attending. Also some patients do not want to attend due to traffic into the city. |
| Having the time and personnel. Central Office continues to remove appointment requirements for established patients and now are working on all veterans having direct access to Audiology without having to go to PCP first. We're getting swamped with regular clinic. |
| Time |
| Staff limitations (only 1 audiologist, 1 tech) |
| Additional time needed by the Audiologists and schedulers for counseling and scheduling specific to the veteran who is complaining of tinnitus and explanation of how the PTM program works.. |
| Lack of space and staff. |
| Do not feel comfortable doing PTM. |

PTM = Progressive Tinnitus Management

CBOC = Community-Based Outpatient Clinic

EES = Employee Education System

PC = Primary Care

ENT = Ear, Nose and Throat

PCP = Primary Care Physician

| ***Mental Health Survey responses*** |
| --- |
| Provider training |
| Under diagnosed. Not on most peoples (sic) radar screen. |
| There does not appeare (sic) to be an awareness outside of the Veterans who experience the issue. |
| Having trained staff. |
| no staff specifically trained in tinnitus protocols, many staff not aware that there are effective protocols |
| Lack of training for psychologists. Lack of knowledge of PCPs that MH can provide interventions. |
| we don't use it on a regular basis due to lack of trained clinicians in this area. |
| training, consultation, and time |
| No training. |
| Not aware of treatment options. Frequency notching to treat tinnitus was something that interested me in the past, but it was not used here. Notching remains of interest to me. |
| Need for recognition and acceptance of the importance of deliberate integration of Mental Health services with the ENT program;At (sic) this point, I am aware of no specific requests for MH interventions in patients with tinnitus. there will be a need for personnel todevelop (sic) expertise in MH management of tinnitus. To ensure success, it ould (sic) be important for the tinnitus services to budget for the MH FTE up front as part of a comprehensive care program, rather than budgeting for all other relevant services except the MH component. |
| not sure |
| Providers do not see tinnitus as a psychiatric disorder |
| limited education |
| Rsources (sic). |
| Lack of training on providing tinnitus services |
| Establishing clinician in a specfic (sic) area of the medical center to offer these services. I do not think general mental health is an appropriate service to provide these services |
| Marketing to/from diverse clinics, as most referrals are coming directly from the Audiology clinic. |
| No problem but, should be handled in a more specialized service for tinnitus like Audiology |
| distance to specialist |
| WIth (sic) the younger Veterans coming back with tinnitus, it has been hard to retain them for tinnitus treatment. There seems to be a lot of interest in tinnitus services but then the turn out for programs can be low overall. |
| Veterans would receive (sic) treatment. |
| Don't know |
| It is a bit esoteric at this point, low awareness and low focus, although it seems it could be very helpful. |
| commitment to a psychotherapeutic inteegrative (sic) approach with full cooperation by Audiology staff, perhaps group space needs as well. |
| training and program development |
| Help the patient's (sic) understand the etiology and help them cope with the constant ringing. |
| Not a lot of information, Veterans seem to prefer medical management of tinnitus |
| Under staffing Lack of Training Excessive workload |
| It is not considered as critical as other major health conditions, but certainly mertis (sic) treatment. |
| 1. Consistent group room availability. 2. There are not enough mental health providers with knowledge and/or clinical time to implement behavioral health practices to treat tinnitus. |
| MH workers are unfamiliar with this intervention |
| training, and allowing time in the established clinics for this type of therapy. |
| no specific training |
| Not sure |
| This survey is WAY TOO Long and assumes a MUCH higher interest in tinnitus than I have. |
| no interest no training |
| Time to leave daily work for training. |
| Provider education/expertise |
| space |
| LAck (sic) of volume lack of expertise |
| 1. Getting a buy-in from administration. I started working with an audiologist and we ran a few PTM workshops. However, I was taken out of this role due to need to focus on other areas. 2. There's limited awareness of this area among staff. I do seminars for interns and postdocs on this topic and also bring in the audiologist. 3. The audiologist's hours in running the program have been decreased by his supervisors. Most of his work is done on his own time. He is also retiring and I haven't seen an investment in the rest of the audiology department in continuing these servies (sic). |
| lack of training |
| Space for holding groups. Scheduling and responding to consults without administrative support. Coordinating care with audiologists. Generating interest and input from audiologists. Getting audiologists to make referrals or to discuss tinnitus with pts due to time constraints. |
| Lack of awareness/acknowledgement of the seriousness of the condition |
| To me the concept that tinnitus should be handled in a mental health clinic seems a little bit of a stretch and it would be better to have a tinnitus program in primary care. |
| ??? |
| lack of knowledge about it |
| training and screening questions |

PCP = Primary Care Physician

MH = Mental Health

ENT = Ear, Nose and Throat

FTE = Full-Time Equivalent

***Following a question about PTM Level 4 Interdisciplinary Evaluation (Q27d. in Audiology Survey), participants were asked who performs the evaluation. If ‘Other’ was selected, participants are given an opportunity to write in a response.***

| ***Audiology Survey response*** |
| --- |
| Audiologist and referral to mental health determined collaboratively |

PTM = Progressive Tinnitus Management

***Following a question about PTM Level 5 Individualized Support (Q27e. in Audiology Survey, Q26e. in Mental Health Survey), participants were asked what services are offered for Level 5. If ‘Other’ was selected, participants are given an opportunity to write in a response.***

| ***Audiology Survey responses*** |
| --- |
| Individual counseling with audiologist or psychologist, Neuromonics, and/or TRT principles |
| I try to follow the PTM program as much as I can but I cannot say the same of my co-workers. I took the time to give them questionnaired (sic) and write down their outcome. I counsel them. Explained every detail of the book. They come to the clinic with the expectations they need a couple of intervews (sic) and convince them they have to work on their (sic) own.. The (sic) don't do the job of the PTM booklet. (I need more books by the way). I spent some time with them to make sure what are their intentions. |

PTM = Progressive Tinnitus Management

TRT = Tinnitus Retraining Therapy

| ***Mental Health Survey responses*** |
| --- |
| A mindfulness [multi-] week program has been offered for several consecutive years. We work closely with auidlogy (sic) and offer services as needed by the Veteran , whether they be individual or group. ALthough (sic) we do not use PTM our model is closely alligned (sic) with the proinciples (sic) of providing education, working closely with audiology and providing more intensive services to those in need. |
| Referral to general mental health clinic for further assessment and services. Primarily groups are available with Veterans able to request one-on-one services if desired. |

PTM = Progressive Tinnitus Management
